# Crystal structure of K_6_[Zn(CO_3_)_4_]

**DOI:** 10.1107/S2056989023006072

**Published:** 2023-07-14

**Authors:** Felix Eder, Matthias Weil

**Affiliations:** a Institute for Chemical Technologies and Analytics, Division of Structural Chemistry, TU Wien, Getreidemarkt 9/E164-05-1, A-1060 Vienna, Austria; University of Kentucky, USA

**Keywords:** crystal structure, zinc in tetra­hedral coordination, carbonate

## Abstract

The crystal structure of K_6_[Zn(CO_3_)_4_], hexa­potassium tetra­carbonato­zincate(II), comprises four unique potassium cations (two located on a general position, and two on the twofold rotation axis of the space group *C*2/*c*) and a [Zn(CO_3_)_4_]^6−^ anion. The Zn^II^ atom of the latter is located on the twofold rotation axis and is surrounded in a slightly distorted tetra­hedral manner by two pairs of monodentately binding carbonate groups, with Zn—O distances of 1.9554 (18) and 1.9839 (18) Å. Both carbonate groups exhibit a slight deviation from planarity, with the C atom being shifted by 0.008 (2) and 0.006 (3) Å, respectively, from the plane of the three O atoms. The coordination numbers of the potassium cations range from 6 to 8, using a threshold of 3.0 Å for K—O bonding inter­actions being significant. In the crystal structure, [KO_*x*_] polyhedra and [Zn(CO_3_)_4_]^6−^ groups share O atoms to build up the framework structure.

## Abstract 2

The crystal structure of the title compound comprises a tetra­carbonatozincate(II) anion, [Zn(CO_3_)_4_]^6−^, with point-group symmetry 2 where the Zn^II^ atom is surrounded in a distorted tetra­hedral manner by four monodentate carbonate groups.

## 1. Chemical context

Oxidotellurates(IV) exhibit a multifarious crystal chemistry (Christy *et al.*, 2016 ▸) that can be attributed to the different coordination numbers of Te^IV^ (usually between 3 and 5) in an oxidic environment and, particularly, to the stereoactive non-bonding 5*s^2^* electron lone pair at the Te^IV^ atom (Galy *et al.*, 1975 ▸). The space requirement of the lone pair leads to unilateral coordination polyhedra [Te^IV^O_*x*_] with rather low point-group symmetries. From a crystal-engineering point of view, [Te^IV^O_*x*_] units are promising building blocks for the construction of new ferro-, pyro- or piezoelectric compounds or materials exhibiting non-linear optical behaviour like second-harmonic generation, as such compounds need to crystallize in non-centrosymmetric space groups with polar axes (Ok *et al.*, 2006 ▸).

In the quest to obtain new transition-metal oxidotellurates(IV) modified by addition of alkali cations, we developed syntheses under pseudo-hydro­thermal conditions where water does not act as a typical solvent but rather as a mineralizer (Eder & Weil, 2022 ▸; Eder *et al.*, 2022 ▸, 2023 ▸). Characteristic for this kind of preparation method, only a few drops of water are added to the reaction mixture instead of the few millilitres typically used in a hydro­thermal experiment. In an alternative route employed also for the present study, water is not added at all to the reaction mixture but originates from the initial decomposition of one of the educt(s) in the closed reaction container where it then acts as a mineralizing agent. Simultaneously, the employed oxidotellurate(VI) phase can be reduced under these conditions to an oxidotellurate(IV). In this sense, solid K_2_CO_3_, ZnO and H_6_TeO_6_ (as the source for water) were treated thermally under these conditions. However, the reaction did not result in an intended potassium zinc oxidotellurate(IV) phase. Instead, K_6_[Zn(CO_3_)_4_] was one of the obtained products, and its crystal structure is reported in the present communication.

## 2. Structural commentary

**Figure 1:**
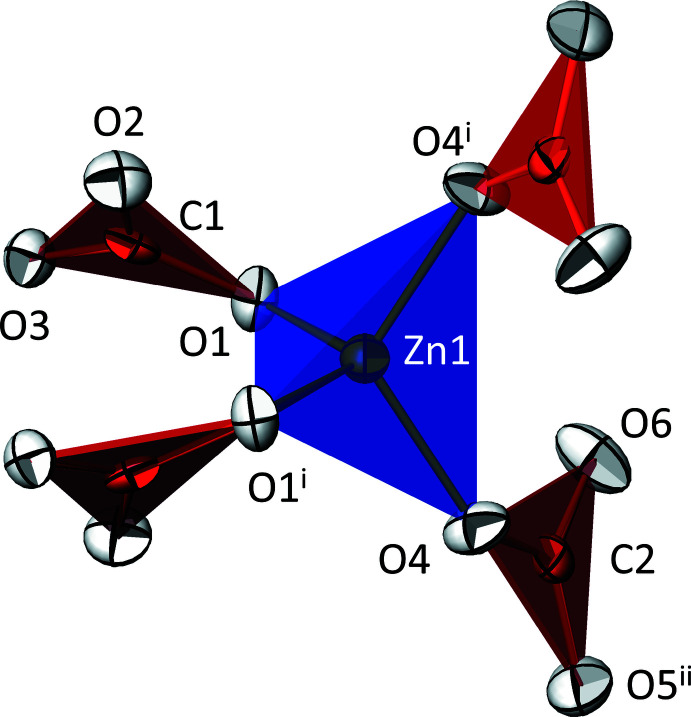
The tetra­hedral [Zn(CO_3_)_4_]^6−^ anion in the crystal structure of K_6_[Zn(CO_3_)_4_], with displacement ellipsoids drawn at the 74% probability level. [Symmetry codes: (i) −*x*, *y*, −*z* + 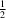 ; (ii) −*x* + 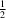 , −*y* + 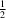 , −*z* + 1.]

Of the 13 atoms (4 K, 1 Zn, 2 C, 6 O) in the asymmetric unit of K_6_[Zn(CO_3_)_4_], three are located on the twofold rotation axis (Zn1, K3, K4; Wyckoff position 4 *e*) of the space group *C*2/*c*. The remaining ten all are located on the general 8 *f* position. The most peculiar structural feature in the crystal structure is the tetra­carbonatozincate(II) anion, [Zn(CO_3_)_4_]^6−^, for which bond lengths and angles are given in Table 1 ▸. The Zn^II^ atom is surrounded in a slightly distorted tetra­hedral manner by two pairs of monodentately binding carbonate groups (Fig. 1 ▸). The mean Zn—O distance of 1.976 Å conforms with the value of 1.952 (31) Å for Zn with a coordination number (CN) of 4 (Gagné & Hawthorne, 2020 ▸). The deviation from the ideal tetra­hedral shape is small (Table 1 ▸), as indicated by the τ_4_ index of 0.92 (τ_4_ = 1 for an ideal tetra­hedron; Yang *et al.*, 2007 ▸). In the carbonate groups, the mean C—O bond lengths of 1.290 (25) Å for C1 and 1.285 (25) Å for C2 are in very good agreement with the grand mean bond length of 1.284 (20) Å calculated from 389 individual carbonate groups (Gagné & Hawthorne, 2018 ▸). In the title compound, the longest C—O bond of ≃ 1.315 Å occurs for the O atoms that are bonded to the Zn^II^ atom. The angular distortions of the carbonate groups are minute (Table 1 ▸), with an angular sum of 360° in each case. However, both CO_3_^2−^ groups in the [Zn(CO_3_)_4_]^6−^ anion are aplanar, with the C atoms slightly shifted out of the plane of the three O atoms [C1 by −0.008 (2) Å from the plane defined by O1, O2, O3 and C2 by −0.006 (3) Å from O4, O5, O6]. Such a deviation from planarity is a frequently observed phenomenon for carbonate groups (Zemann, 1981 ▸; Winkler *et al.*, 2000 ▸).

The charge of the [Zn(CO_3_)_4_]^6−^ anion is compensated by large potassium cations. Since coordination numbers of large cations are not always simple to derive because there is no clear boundary for longer bonds and the corresponding (weak) inter­actions between the central atom and the ligand atom (Gagné & Hawthorne, 2016 ▸), we defined a threshold of 3.0 Å for K—O inter­actions as being significant in K_6_[Zn(CO_3_)_4_]. Based on this value, K1 and K2 have a CN of 7, K3 of 8 and K4 of 6, with distorted [KO_*x*_] polyhedra in each case. The mean K—O bond lengths of 2.852 Å (K1), 2.763 Å (K2), 2.809 Å (K3) and 2.814 Å (K4) roughly correlate with literature values (Gagné & Hawthorne, 2016 ▸) of 2.828 (177) Å for a CN of 6, 2.861 (179) Å for a CN of 7, and 2.894 (172) Å for a CN of 8. The large standard deviations of the literature data likewise reflect the difficulties in defining coordination numbers for large cations.

Bond-valence sums (Brown, 2002 ▸) were calculated with the values provided by Brese & O’Keeffe (1991 ▸). Individual values (in valence units) are collated in the following list and are in agreement with the expected values of 1 for K, 2 for Zn, 4 for C and 2 for O: K1: 1.02; K2: 1.31; K3: 1.32; K4: 0.96; Zn1: 1.95; C1: 3.84; C2: 3.99; O1 (CN = 4 with C, Zn, 2K): 1.94; O2 (CN = 5 with C, 4K): 1.92; O3 (CN = 6 with C, 5K): 2.18; O4 (CN = 4 with C, Zn, 2K): 2.00; O5 (CN = 5 with C, 4K): 1.92; O6 (CN = 5 with C, 4K): 1.92.

**Figure 2:**
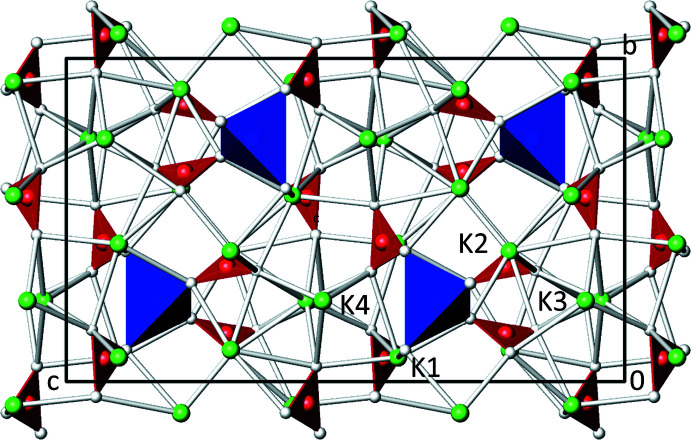
The crystal structure of K_6_[Zn(CO_3_)_4_] in a projection along [ 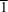 00]. Carbonate groups are shown as flattened red polyhedra and [ZnO_4_] units as blue tetra­hedra. All atoms are drawn as spheres of arbitrary radii (K green, O white, Zn blue, C red).

In the crystal structure of K_6_[Zn(CO_3_)_4_], [KO_*x*_] polyhedra and the isolated [Zn(CO_3_)_4_]^6−^ anions share O atoms to build up a framework (Fig. 2 ▸).

## 3. Database survey

A search in the Inorganic Structure Database (ICSD, version April 2022; Zagorac *et al.*, 2019 ▸) for mixed alkali-metal/trans­ition-metal carbonates revealed only eight anhydrous phases, *viz*. Na_2_Cu(CO_3_)_2_ (Healy & White, 1972 ▸), K_2_Cu(CO_3_)_2_ (Farrand *et al.*, 1980 ▸), Na_3_Y(CO_3_)_3_ (Luo *et al.*, 2014 ▸), Na_5_Y(CO_3_)_4_ (Awaleh *et al.*, 2003 ▸), KY(CO_3_)_2_ (Cao *et al.*, 2018 ▸), Na_2_Cd(CO_3_)_2_ (Kim *et al.*, 2018 ▸), K_2_Cd(CO_3_)_2_ (Kim *et al.*, 2021 ▸), and KAgCO_3_ (Hans *et al.*, 2015 ▸). This makes K_6_[Zn(CO_3_)_4_] the phase with the highest qu­antity of an alkali metal. Except for the two copper(II) compounds where Cu^II^ shows a square-planar coordination by carbonate O atoms, the coordination numbers of all other transition metals are higher than 4.

However, numerous hydrous mixed alkali-metal/transition-metal carbonates are known. Limited to mixed alkali-metal zinc carbonates, these are: LiZn(CO_3_)(OH) (Liu *et al.*, 2021 ▸), NaZn(CO_3_)(OH) (Peng *et al.*, 2020 ▸), Na_2_Zn_3_(CO_3_)_4_·3H_2_O (Gier *et al.*, 1996 ▸), NaK_2_{Zn_2_[H(CO_3_)_2_](CO_3_)_2_(H_2_O)_2_ and NaRb_2_{Zn_2_[H(CO_3_)_2_](CO_3_)_2_(H_2_O)_2_ (Zheng & Adam, 1995 ▸).

In the crystal structure of LiZn(CO_3_)(OH), the Zn^II^ atom is tetra­hedrally coordinated by two O atoms of monodentate carbonate groups and two bridging OH groups, leading to _∞_^1^[ZnO_2/1_(OH)_2/2_] chains extending parallel to [100] that are bridged by the carbonate groups into layers. In NaZn(CO_3_)(OH), the Zn^II^ atom is likewise tetra­hedrally coordinated by two O atoms of monodentate carbonate groups and two OH groups, leading to isolated [ZnO_2_(OH)_2_] tetra­hedra. In the crystal structure of Na_2_Zn_3_(CO_3_)_4_·3H_2_O, the Zn^II^ atom is coordinated tetra­hedrally by four oxygen atoms belonging to four carbonate ions. Each carbonate group binds to three different zinc atoms forming an open framework structure. Finally, in NaK_2_{Zn_2_[H(CO_3_)_2_](CO_3_)_2_(H_2_O)_2_ and isotypic NaRb_2_{Zn_2_[H(CO_3_)_2_](CO_3_)_2_(H_2_O)_2_, the Zn^II^ atom is coord­inated by five oxygen atoms belonging to four carbonate groups and one water mol­ecule. Very similarly, in Na_3_Zn_2_(CO_3_)_3_F (Tang *et al.*, 2018 ▸) the same coordination number results by coordination from four carbonate groups and a fluoride anion.

## 4. Synthesis and crystallization

All employed educts were obtained from commercial sources and were chemically pure. Solid ZnO, H_6_TeO_6_ and K_2_CO_3_ were thoroughly mixed in the molar ratio 2:3:10 (original sample weights 0.0584 g, 0.2486 g, 0.4498 g, respectively) and locked in a Teflon container with an inner volume of about 3 ml. The container was sealed and placed in a steel autoclave that was heated for one week at 483 K. The obtained solid product was colourless, comprising the title compound in the form of a few colourless crystals with a plate-like form. Powder X-ray diffraction (PXRD) revealed K_6_[Zn(CO_3_)_4_], K_2_CO_3_·1.5H_2_O (Skakle *et al.*, 2001 ▸), KTeO_3_OH (Lindqvist, 1972 ▸) and the starting material ZnO as product phases with approximate contingents (in mass percentages) of 45%, 40%, 10% and <5%, respectively, together with some unassigned reflections of low intensities.

K_6_[Zn(CO_3_)_4_] could also be synthesized by slow evaporation of a solution containing Zn(NO_3_)_2_·6H_2_O and K_2_CO_3_ in a molar ratio of 1:5, resulting in an increased yield of the title compound (70%), together with K_2_CO_3_·1.5H_2_O (25%) and ZnO (<5%) as by-products, as determined by phase analysis on basis of PXRD data.

## 5. Refinement

Crystal data, data collection and structure refinement details are summarized in Table 2 ▸. Structure data were standardized with *STRUCTURE-TIDY* (Gelato & Parthé, 1987 ▸).

## Supplementary Material

Crystal structure: contains datablock(s) I. DOI: 10.1107/S2056989023006072/pk2691sup1.cif

Structure factors: contains datablock(s) I. DOI: 10.1107/S2056989023006072/pk2691Isup3.hkl

CCDC reference: 2280530

Additional supporting information:  crystallographic information; 3D view; checkCIF report

## Figures and Tables

**Table 1:** Selected geometric parameters (Å, °)

Zn1—O4	1.9554 (18)	O2—C1	1.273 (3)
Zn1—O4^i^	1.9554 (18)	O3—C1	1.278 (3)
Zn1—O1^i^	1.9838 (18)	O4—C2	1.313 (3)
Zn1—O1	1.9839 (18)	O5—C2^ii^	1.268 (3)
O1—C1	1.319 (3)	O6—C2	1.273 (3)
			
O4—Zn1—O4^i^	113.95 (11)	O2—C1—O1	120.1 (2)
O4—Zn1—O1^i^	99.62 (8)	O3—C1—O1	118.3 (2)
O4—Zn1—O1	114.01 (8)	O5^ii^—C2—O6	123.1 (3)
O1^i^—Zn1—O1	116.44 (10)	O5^ii^—C2—O4	118.0 (2)
O2—C1—O3	121.7 (2)	O6—C2—O4	119.0 (2)

Symmetry codes: (i) 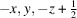 ; (ii) 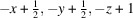 .

**Table 2:** Experimental details

Crystal data	
Chemical formula	K_6_[Zn(CO_3_)_4_]
*M*_r_	540.01
Crystal system, space group	Monoclinic, *C*2/*c*
Temperature (K)	296
*a*, *b*, *c* (Å)	7.1850 (6), 18.1117 (14), 10.5206 (8)
β (°)	93.579 (2)
*V* (Å^3^)	1366.40 (19)
*Z*	4
Radiation type	Mo *K*α
μ (mm^−1^)	3.69
Crystal size (mm)	0.08 × 0.04 × 0.02

Data collection	
Diffractometer	Bruker APEXII CCD
Absorption correction	Multi-scan (*SADABS*; Krause *et al.*, 2015 ▸)
*T*_min_, *T*_max_	0.665, 0.747
No. of measured, independent and observed [*I* > 2σ(*I*)] reflections	8980, 2592, 1712
*R*_int_	0.056
(sin θ/λ)_max_ (Å^−1^)	0.785

Refinement	
*R*[*F* ^2^ > 2σ(*F* ^2^)], *wR*(*F* ^2^), *S*	0.040, 0.072, 0.98
No. of reflections	2592
No. of parameters	106
Δρ_max_, Δρ_min_ (e Å^−3^)	1.02, −0.63

Computer programs: *APEX3* and *SAINT* (Bruker, 2016 ▸), *SHELXT* (Sheldrick, 2015*a* ▸), *SHELXL* (Sheldrick, 2015*b* ▸), *ATOMS* (Dowty, 2006 ▸), *PLATON* (Spek, 2020 ▸) and *publCIF* (Westrip, 2010 ▸).

## supplementary crystallographic information

### Crystal data

**Table:**

K_6_[Zn(CO_3_)_4_]	*F*(000) = 1056
*M**_r_* = 540.01	*D*_x_ = 2.625 Mg m^−^^3^
Monoclinic, *C*2/*c*	Mo *K*α radiation, λ = 0.71073 Å
*a* = 7.1850 (6) Å	Cell parameters from 1545 reflections
*b* = 18.1117 (14) Å	θ = 4.5–29.4°
*c* = 10.5206 (8) Å	µ = 3.69 mm^−^^1^
β = 93.579 (2)°	*T* = 296 K
*V* = 1366.40 (19) Å^3^	Block, colourless
*Z* = 4	0.08 × 0.04 × 0.02 mm

### Data collection

**Table:**

Bruker APEXII CCD diffractometer	1712 reflections with *I* > 2σ(*I*)
ω– and φ–scans	*R*_int_ = 0.056
Absorption correction: multi-scan (*SADABS*; Krause *et al.*, 2015)	θ_max_ = 33.9°, θ_min_ = 3.0°
*T*_min_ = 0.665, *T*_max_ = 0.747	*h* = −11→11
8980 measured reflections	*k* = −26→27
2592 independent reflections	*l* = −15→16

### Refinement

**Table:**

Refinement on *F*^2^	106 parameters
Least-squares matrix: full	0 restraints
*R*[*F*^2^ > 2σ(*F*^2^)] = 0.040	*w* = 1/[σ^2^(*F*_o_^2^) + (0.0241*P*)^2^] where *P* = (*F*_o_^2^ + 2*F*_c_^2^)/3
*wR*(*F*^2^) = 0.072	(Δ/σ)_max_ = 0.001
*S* = 0.98	Δρ_max_ = 1.02 e Å^−^^3^
2592 reflections	Δρ_min_ = −0.63 e Å^−^^3^

### Special details

**Table:**

Geometry. All esds (except the esd in the dihedral angle between two l.s. planes) are estimated using the full covariance matrix. The cell esds are taken into account individually in the estimation of esds in distances, angles and torsion angles; correlations between esds in cell parameters are only used when they are defined by crystal symmetry. An approximate (isotropic) treatment of cell esds is used for estimating esds involving l.s. planes.

### Fractional atomic coordinates and isotropic or equivalent isotropic displacement parameters (Å^2^)

**Table:**

	*x*	*y*	*z*	*U*_iso_*/*U*_eq_	
Zn1	0.000000	0.33426 (2)	0.250000	0.01451 (11)	
K1	0.34220 (9)	0.40604 (3)	0.07452 (6)	0.02200 (14)	
K2	0.38485 (8)	0.20542 (3)	0.40224 (6)	0.02155 (14)	
K3	0.000000	0.06967 (4)	0.250000	0.01697 (17)	
K4	0.000000	0.54036 (5)	0.250000	0.0235 (2)	
O1	0.2160 (3)	0.27658 (10)	0.19578 (18)	0.0184 (4)	
O2	0.0052 (3)	0.20449 (10)	0.08928 (18)	0.0216 (4)	
O3	0.2802 (2)	0.15837 (9)	0.15907 (16)	0.0169 (4)	
O4	0.0538 (3)	0.39310 (10)	0.40374 (17)	0.0206 (4)	
O5	0.2533 (3)	0.05284 (11)	0.45294 (18)	0.0264 (5)	
O6	0.3006 (3)	0.45433 (11)	0.34053 (19)	0.0271 (5)	
C1	0.1656 (4)	0.21218 (14)	0.1462 (2)	0.0146 (5)	
C2	0.2048 (4)	0.43226 (13)	0.4310 (2)	0.0155 (5)	

### Atomic displacement parameters (Å^2^)

**Table:**

	*U*^11^	*U*^22^	*U*^33^	*U*^12^	*U*^13^	*U*^23^
Zn1	0.0148 (2)	0.0142 (2)	0.0144 (2)	0.000	−0.00042 (17)	0.000
K1	0.0222 (3)	0.0204 (3)	0.0233 (3)	−0.0016 (2)	0.0010 (3)	−0.0040 (2)
K2	0.0210 (3)	0.0265 (3)	0.0168 (3)	0.0032 (3)	−0.0013 (2)	−0.0030 (2)
K3	0.0162 (4)	0.0160 (4)	0.0188 (4)	0.000	0.0014 (3)	0.000
K4	0.0177 (4)	0.0182 (4)	0.0339 (5)	0.000	−0.0038 (4)	0.000
O1	0.0173 (10)	0.0130 (8)	0.0250 (10)	−0.0008 (8)	0.0026 (8)	−0.0020 (8)
O2	0.0163 (9)	0.0263 (11)	0.0217 (10)	0.0018 (8)	−0.0035 (8)	0.0007 (8)
O3	0.0146 (9)	0.0161 (9)	0.0200 (10)	0.0034 (7)	0.0012 (8)	−0.0006 (7)
O4	0.0189 (10)	0.0259 (10)	0.0169 (10)	−0.0088 (8)	0.0005 (8)	−0.0049 (8)
O5	0.0317 (12)	0.0246 (10)	0.0213 (10)	−0.0024 (9)	−0.0100 (9)	−0.0034 (9)
O6	0.0193 (10)	0.0332 (12)	0.0290 (12)	−0.0050 (9)	0.0018 (9)	0.0138 (9)
C1	0.0152 (12)	0.0191 (13)	0.0097 (12)	0.0012 (10)	0.0022 (10)	0.0048 (10)
C2	0.0185 (13)	0.0128 (12)	0.0149 (13)	0.0029 (10)	−0.0028 (11)	0.0026 (10)

### Geometric parameters (Å, º)

**Table:**

Zn1—O4	1.9554 (18)	K3—O5	2.7344 (19)
Zn1—O4^i^	1.9554 (18)	K3—O6^ix^	2.738 (2)
Zn1—O1^i^	1.9838 (18)	K3—O6^x^	2.738 (2)
Zn1—O1	1.9839 (18)	K3—O3	2.7910 (18)
K1—O5^ii^	2.756 (2)	K3—O3^i^	2.7910 (18)
K1—O6^iii^	2.804 (2)	K3—O2^i^	2.972 (2)
K1—O3^iv^	2.8113 (18)	K3—O2	2.972 (2)
K1—O1	2.8448 (19)	K3—C1^i^	3.071 (3)
K1—O4^i^	2.879 (2)	K3—C1	3.071 (3)
K1—O2^iv^	2.901 (2)	K3—K4^ix^	3.6315 (3)
K1—O6	2.965 (2)	K3—K4^xi^	3.6315 (3)
K1—C1^iv^	3.156 (3)	K4—O6^i^	2.782 (2)
K1—C2^iii^	3.293 (3)	K4—O6	2.783 (2)
K1—O5^v^	3.374 (2)	K4—O3^ii^	2.7908 (18)
K1—C2^vi^	3.412 (3)	K4—O3^xii^	2.7908 (18)
K2—O2^vii^	2.6590 (19)	K4—O5^xii^	2.868 (2)
K2—O3^iii^	2.6697 (19)	K4—O5^ii^	2.868 (2)
K2—O4^viii^	2.726 (2)	K4—C2	3.046 (2)
K2—O1	2.7426 (19)	K4—C2^i^	3.046 (2)
K2—O3	2.7561 (18)	K4—O4^i^	3.131 (2)
K2—O2^i^	2.810 (2)	K4—O4	3.131 (2)
K2—O5	2.980 (2)	O1—C1	1.319 (3)
K2—C1	3.037 (3)	O2—C1	1.273 (3)
K2—C2^viii^	3.139 (3)	O3—C1	1.278 (3)
K2—C1^iii^	3.303 (3)	O4—C2	1.313 (3)
K2—K2^viii^	3.3304 (12)	O5—C2^viii^	1.268 (3)
K2—O1^iii^	3.3644 (19)	O6—C2	1.273 (3)
K3—O5^i^	2.7344 (19)		
			
O4—Zn1—O4^i^	113.95 (11)	O6^i^—K4—O5^xii^	78.24 (6)
O4—Zn1—O1^i^	99.62 (8)	O6—K4—O5^xii^	106.98 (6)
O4^i^—Zn1—O1^i^	114.01 (8)	O3^ii^—K4—O5^xii^	92.71 (5)
O4—Zn1—O1	114.01 (8)	O3^xii^—K4—O5^xii^	80.33 (5)
O4^i^—Zn1—O1	99.62 (8)	O6^i^—K4—O5^ii^	106.98 (6)
O1^i^—Zn1—O1	116.44 (10)	O6—K4—O5^ii^	78.24 (6)
O5^ii^—K1—O6^iii^	87.09 (6)	O3^ii^—K4—O5^ii^	80.33 (5)
O5^ii^—K1—O3^iv^	104.27 (6)	O3^xii^—K4—O5^ii^	92.71 (5)
O6^iii^—K1—O3^iv^	129.66 (6)	O5^xii^—K4—O5^ii^	170.96 (8)
O5^ii^—K1—O1	139.27 (6)	O6^i^—K4—O4^i^	43.77 (5)
O6^iii^—K1—O1	115.16 (6)	O6—K4—O4^i^	76.59 (6)
O3^iv^—K1—O1	87.64 (5)	O3^ii^—K4—O4^i^	151.47 (5)
O5^ii^—K1—O4^i^	81.15 (6)	O3^xii^—K4—O4^i^	115.23 (5)
O6^iii^—K1—O4^i^	152.83 (6)	O5^xii^—K4—O4^i^	112.93 (6)
O3^iv^—K1—O4^i^	77.22 (5)	O5^ii^—K4—O4^i^	75.21 (5)
O1—K1—O4^i^	63.44 (5)	C2—K4—O4^i^	79.32 (6)
O5^ii^—K1—O2^iv^	134.76 (6)	C2^i^—K4—O4^i^	24.49 (6)
O6^iii^—K1—O2^iv^	91.85 (6)	O6^i^—K4—O4	76.59 (6)
O3^iv^—K1—O2^iv^	45.86 (5)	O6—K4—O4	43.77 (5)
O1—K1—O2^iv^	80.82 (5)	O3^ii^—K4—O4	115.23 (5)
O4^i^—K1—O2^iv^	113.78 (6)	O3^xii^—K4—O4	151.47 (5)
O5^ii^—K1—O6	77.01 (6)	O5^xii^—K4—O4	75.21 (5)
O6^iii^—K1—O6	75.60 (6)	O5^ii^—K4—O4	112.93 (6)
O3^iv^—K1—O6	154.55 (6)	C2—K4—O4	24.49 (6)
O1—K1—O6	76.47 (5)	C2^i^—K4—O4	79.32 (6)
O4^i^—K1—O6	77.91 (6)	O4^i^—K4—O4	63.16 (7)
O2^iv^—K1—O6	145.94 (6)	C1—O1—Zn1	112.29 (16)
O5^ii^—K1—O5^v^	88.01 (6)	C1—O1—K2	89.70 (14)
O6^iii^—K1—O5^v^	41.12 (5)	Zn1—O1—K2	109.58 (8)
O3^iv^—K1—O5^v^	89.64 (5)	C1—O1—K1	129.69 (16)
O1—K1—O5^v^	131.53 (5)	Zn1—O1—K1	88.39 (6)
O4^i^—K1—O5^v^	160.24 (5)	K2—O1—K1	127.22 (7)
O2^iv^—K1—O5^v^	63.57 (5)	C1—O1—K2^iii^	76.00 (13)
O6—K1—O5^v^	115.78 (5)	Zn1—O1—K2^iii^	170.74 (8)
C1^iv^—K1—O5^v^	81.18 (6)	K2—O1—K2^iii^	73.70 (5)
C2^iii^—K1—O5^v^	21.89 (5)	K1—O1—K2^iii^	82.91 (5)
O2^vii^—K2—O3^iii^	96.79 (6)	C1—O2—K2^xiii^	121.60 (16)
O2^vii^—K2—O4^viii^	79.50 (6)	C1—O2—K2^i^	149.34 (17)
O3^iii^—K2—O4^viii^	82.30 (6)	K2^xiii^—O2—K2^i^	74.98 (5)
O2^vii^—K2—O1	113.90 (6)	C1—O2—K1^iv^	89.44 (15)
O3^iii^—K2—O1	108.64 (6)	K2^xiii^—O2—K1^iv^	95.82 (6)
O4^viii^—K2—O1	160.62 (6)	K2^i^—O2—K1^iv^	115.96 (7)
O2^vii^—K2—O3	159.06 (6)	C1—O2—K3	82.28 (14)
O3^iii^—K2—O3	82.81 (6)	K2^xiii^—O2—K3	155.54 (8)
O4^viii^—K2—O3	120.97 (6)	K2^i^—O2—K3	86.49 (5)
O1—K2—O3	47.83 (5)	K1^iv^—O2—K3	77.86 (5)
O2^vii^—K2—O2^i^	105.02 (5)	C1—O3—K2^iii^	108.39 (15)
O3^iii^—K2—O2^i^	157.18 (6)	C1—O3—K2	89.96 (14)
O4^viii^—K2—O2^i^	95.00 (6)	K2^iii^—O3—K2	85.86 (5)
O1—K2—O2^i^	68.60 (5)	C1—O3—K4^xi^	165.41 (16)
O3—K2—O2^i^	79.21 (6)	K2^iii^—O3—K4^xi^	80.09 (5)
O2^vii^—K2—O5	121.93 (6)	K2—O3—K4^xi^	78.63 (5)
O3^iii^—K2—O5	92.76 (6)	C1—O3—K3	90.09 (15)
O4^viii^—K2—O5	45.34 (5)	K2^iii^—O3—K3	161.25 (7)
O1—K2—O5	116.61 (6)	K2—O3—K3	91.18 (5)
O3—K2—O5	78.94 (5)	K4^xi^—O3—K3	81.17 (5)
O2^i^—K2—O5	70.15 (6)	C1—O3—K1^iv^	93.41 (14)
O2^vii^—K2—O1^iii^	75.38 (5)	K2^iii^—O3—K1^iv^	99.14 (6)
O3^iii^—K2—O1^iii^	41.38 (5)	K2—O3—K1^iv^	172.75 (7)
O4^viii^—K2—O1^iii^	112.29 (5)	K4^xi^—O3—K1^iv^	96.97 (5)
O1—K2—O1^iii^	85.45 (6)	K3—O3—K1^iv^	82.40 (5)
O3—K2—O1^iii^	91.28 (5)	C2—O4—Zn1	126.39 (17)
O2^i^—K2—O1^iii^	152.02 (5)	C2—O4—K2^viii^	95.59 (14)
O5—K2—O1^iii^	134.13 (5)	Zn1—O4—K2^viii^	106.08 (8)
C1—K2—O1^iii^	96.75 (6)	C2—O4—K1^i^	138.26 (16)
C2^viii^—K2—O1^iii^	133.18 (6)	Zn1—O4—K1^i^	87.97 (7)
C1^iii^—K2—O1^iii^	22.80 (5)	K2^viii^—O4—K1^i^	96.21 (6)
K2^viii^—K2—O1^iii^	122.93 (4)	C2—O4—K4	74.12 (13)
O5^i^—K3—O5	167.20 (9)	Zn1—O4—K4	91.45 (7)
O5^i^—K3—O6^ix^	81.34 (6)	K2^viii^—O4—K4	162.44 (7)
O5—K3—O6^ix^	88.88 (6)	K1^i^—O4—K4	83.15 (5)
O5^i^—K3—O6^x^	88.88 (6)	C2^viii^—O5—K3	146.56 (17)
O5—K3—O6^x^	81.33 (6)	C2^viii^—O5—K1^x^	110.41 (16)
O6^ix^—K3—O6^x^	80.53 (9)	K3—O5—K1^x^	82.91 (6)
O5^i^—K3—O3	104.81 (6)	C2^viii^—O5—K4^xi^	127.79 (17)
O5—K3—O3	82.69 (6)	K3—O5—K4^xi^	80.78 (5)
O6^ix^—K3—O3	164.34 (6)	K1^x^—O5—K4^xi^	90.42 (6)
O6^x^—K3—O3	85.15 (6)	C2^viii^—O5—K2	85.18 (15)
O5^i^—K3—O3^i^	82.69 (6)	K3—O5—K2	87.70 (6)
O5—K3—O3^i^	104.81 (6)	K1^x^—O5—K2	162.84 (8)
O6^ix^—K3—O3^i^	85.16 (6)	K4^xi^—O5—K2	73.86 (5)
O6^x^—K3—O3^i^	164.34 (6)	C2^viii^—O5—K1^xiv^	75.47 (15)
O3—K3—O3^i^	109.72 (7)	K3—O5—K1^xiv^	73.49 (5)
O5^i^—K3—O2^i^	120.30 (6)	K1^x^—O5—K1^xiv^	91.99 (6)
O5—K3—O2^i^	71.26 (6)	K4^xi^—O5—K1^xiv^	153.64 (7)
O6^ix^—K3—O2^i^	113.80 (6)	K2—O5—K1^xiv^	99.10 (6)
O6^x^—K3—O2^i^	148.26 (5)	C2—O6—K3^xv^	144.96 (18)
O3—K3—O2^i^	75.94 (5)	C2—O6—K4	89.28 (15)
O3^i^—K3—O2^i^	45.33 (5)	K3^xv^—O6—K4	82.27 (5)
O5^i^—K3—O2	71.26 (6)	C2—O6—K1^iii^	100.98 (15)
O5—K3—O2	120.30 (6)	K3^xv^—O6—K1^iii^	81.98 (5)
O6^ix^—K3—O2	148.26 (5)	K4—O6—K1^iii^	163.55 (8)
O6^x^—K3—O2	113.80 (6)	C2—O6—K1	134.75 (17)
O3—K3—O2	45.34 (5)	K3^xv^—O6—K1	79.10 (5)
O3^i^—K3—O2	75.94 (5)	K4—O6—K1	87.93 (6)
O2^i^—K3—O2	69.49 (8)	K1^iii^—O6—K1	93.70 (6)
O6^i^—K4—O6	111.90 (9)	O2—C1—O3	121.7 (2)
O6^i^—K4—O3^ii^	163.07 (6)	O2—C1—O1	120.1 (2)
O6—K4—O3^ii^	84.32 (6)	O3—C1—O1	118.3 (2)
O6^i^—K4—O3^xii^	84.32 (6)	O5^viii^—C2—O6	123.1 (3)
O6—K4—O3^xii^	163.07 (6)	O5^viii^—C2—O4	118.0 (2)
O3^ii^—K4—O3^xii^	80.03 (8)	O6—C2—O4	119.0 (2)

Symmetry codes: (i) −*x*, *y*, −*z*+1/2; (ii) −*x*+1/2, *y*+1/2, −*z*+1/2; (iii) −*x*+1, *y*, −*z*+1/2; (iv) −*x*+1/2, −*y*+1/2, −*z*; (v) *x*+1/2, −*y*+1/2, *z*−1/2; (vi) *x*, −*y*+1, *z*−1/2; (vii) *x*+1/2, −*y*+1/2, *z*+1/2; (viii) −*x*+1/2, −*y*+1/2, −*z*+1; (ix) *x*−1/2, *y*−1/2, *z*; (x) −*x*+1/2, *y*−1/2, −*z*+1/2; (xi) *x*+1/2, *y*−1/2, *z*; (xii) *x*−1/2, *y*+1/2, *z*; (xiii) *x*−1/2, −*y*+1/2, *z*−1/2; (xiv) *x*−1/2, −*y*+1/2, *z*+1/2; (xv) *x*+1/2, *y*+1/2, *z*.
